# Letter to Editor: "Combined aerobic and strength training improves dynamic stability and can prevent against static stability decline in postmenopausal women: A randomized clinical trial"

**DOI:** 10.61622/rbgo/2024rbgo26

**Published:** 2024-04-09

**Authors:** Arshdeep Kaur, Amit Kumar

**Affiliations:** 1 Lovely Professional University, Phagwara Punjab India Lovely Professional University, Phagwara, Punjab, India.

Dear Editor,

First and foremost, we express our gratitude towards the authors for their clear and concise description of the positive effects of aerobic and strength training on dynamic stability.^(1)^ Additionally, their ability to provide a focused and informative introduction section is commendable. The study piqued our interest in further exploring the benefits of aerobic and strength training in enhancing balance, posture, and gait patterns in postmenopausal women. We would like to draw attention to a few methodological and statistical issues that are pertinent to the study, as this would enable medical professionals involved in the rehabilitation of postmenopausal women to effectively utilize the study's findings.

According to the guidelines outlined in the journal, the title should not exceed a maximum of 15 words. However, the title in question surpasses this limit and presents confusion for the readers. This is due to the fact that, in the background section, only terms related to posture and gait were utilized. Nevertheless, there exist various parameters that could be incorporated into the concept of dynamic stability, such as balance, equilibrium, and coordination. Another salient aspect that the authors should bear in mind is the necessity for word usage to be well-balanced. One particular inconsistency arises from the utilization of the term "randomized clinical trial" in the title, "control trial" in the abstract, and "controlled" in the methods section. Such discrepancies in terminology can undoubtedly lead to confusion among the readers. Furthermore, in the conclusion section of the abstract, the authors mentioned improved gait and balanced control in older women, which does not align with the objective of the study.

In the third paragraph of the methods section, the authors have provided a detailed account of the reasons for participant dropouts. In order to mitigate this issue, the authors could have employed interim analysis^(2)^ or intention-to-treat analysis,^(3)^ both of which would have enhanced the feasibility of this study. In the section pertaining to sample size estimation, the authors have furnished the values of partial eta square, albeit without any citation. Furthermore, the method employed for determining the sample size remains unspecified, thus potentially leading to confusion. Upon entering all these values into the G* Power 3.1.9.7 software, the estimated sample size was determined to be 952 (with 476 participants in each group), a figure that deviates from the sample size mentioned.

The inclusion of the effect size is essential in order to obtain comprehensive results. Therefore, we have incorporated the effect size values of the outcome measures utilized in the study into the Table. The effect size for the outcome measures was determined using the subsequent formula: [M1 - M2/SDPooled]. The power of the study was assessed using G*Power software ver. 3.1.9.7 (Heinrich-Heine Universitat Dusseldorf, Dusseldorf, Germany; http://www.gpower.hhu.de), which estimated the post hoc power analysis for the effect size of the outcomes.^(4)^ From the table 1, it becomes evident that these measures cannot be adequately discussed due to insufficient power.

**Table 1:** Effect size and power for the measures (Body weight, muscle strength, abdominal, and aerobic capacity)

Measures	Effect size-Control Group	Power-Control Group (%)	Effect size-CT Group)	Power-CT Group (%)
Body weight	0.04	60	0.05	0
Muscle strength	0.57	42	0.29	18
Abdominal	0.23	36	0.75	60
Aerobic capacity	0.07	70	0.74	90
