# Supplementary material for: Letter to Editor: "Combined aerobic and strength training improves dynamic stability and can prevent against static stability decline in postmenopausal women: A randomized clinical trial"
Source: Rev Bras Ginecol Obstet. 2024 Apr 9;46:e-rbgo26. doi: 10.61622/rbgo/2024rbgo26 (PMC11075413; doi:10.61622/rbgo/2024rbgo26)
Supplement: Related texts

# Reply to: Combined aerobic plus strength training improves dynamic stability and can prevent against static stability decline in postmenopausal women: A randomized clinical trial

**Authors:** Ana Claudia Fortaleza Marques, Fabrício Eduardo Rossi, Lucas Melo Neves, Tiego Aparecido Diniz, Iracimara de Anchieta Messias, José Barela, Fay Horak, Ismael Forte Freitas

**Affiliations:** 1 Universidade do Oeste Paulista Department of Medicine SP Brazil Department of Medicine, Universidade do Oeste Paulista, Presidente Prudente, SP, Brazil.; 2 Universidade Federal do Piauí Department of Physical Education Teresina PI Brazil Immunometabolism of Skeletal Muscle and Exercise Research Group, Department of Physical Education, Universidade Federal do Piauí, Teresina, PI, Brazil.; 4 Santo Amaro University São Paulo SP Brazil Santo Amaro University, São Paulo, SP, Brazil.; 4 Universidade de São Paulo Department of Psychiatry São Paulo SP Brazil Department of Psychiatry, Universidade de São Paulo, São Paulo, SP, Brazil.; 5 University of São Paulo São Paulo SP Brazil University of São Paulo, São Paulo, SP Brazil.; 6 Universidade Estadual Paulista "Júlio de Mesquita Filho" Faculty of Science and Technology SP Brazil Faculty of Science and Technology, Universidade Estadual Paulista "Júlio de Mesquita Filho", Presidente Prudente, SP, Brazil.; 7 Instituto de Biociências Department of Physical Education Rio Claro SP Brazil Department of Physical Education, Instituto de Biociências, Rio Claro, SP, Brazil.; 8 Oregon Health & Science University Department of Neurology Portland OR USA Department of Neurology, Oregon Health & Science University, Portland, OR, USA.

Dear Editor,

We read with interest the comments of Kaur and Kumar about the few methodological and statistical issues pertinent to our study "Combined aerobic and strength training improves dynamic stability and can prevent against static stability decline in postmenopausal women: A randomized clinical trial".

In summary, we have demonstrated that Combined Aerobic and Strength Training is safe when administered by trained, experienced, rehabilitation professionals and this training strategy can improve dynamic stability in postmenopausal women. It is very important to highlight that the approved version of our paper was reviewed by the *Revista Brasileira de Ginecologia e Obstetrícia* and the study was registered in the Brazilian database of clinical trials (RBR-9CBP8S).

We believe that our title highlights the importance of the study and included the theme, method of training used, sample and results of our study, as suggested by Woods and Sikes.^(1)^ Furthermore, we understand the importance of different terminologies used in the literature about gait and balance control. In the current study, we choose the terminology "postural control" and "gait performance", since these terms reflect our main objective. Regarding the use of different terms related to "randomized clinical trial", our intent was to use synonyms to prevent word repetition.

Regarding the Methods, the inclusion criteria were postmenopausal women with ages between 50 and 79 on the date of the evaluation. Since the average was 59.3 ± 8.0 years old, we also had participants over 60 yrs old, therefore, older women were included. We understand about the value of intention-to-treat analysis. However, it is prohibited to pay for participants to be rewarded for commitment to clinical trials in Brazil so we have difficultly motivating participants to return to the study after they drop out of the intervention. In addition, the power sample was calculated for the primary outcome (gait velocity). Unfortunately, it would be very difficult and expensive to conduct an exercise intervention study over 16 weeks with 476 participants in each group. Therefore, we have focused the power on the primary outcome. We are confident in our findings and the importance of this study for postmenopausal women.

## References

[1] Woods P, Sikes P (2022). Successful writing for qualitative researchers.
